# Supplementary material for: Differential cellular immune responses against Orientia tsutsugamushi Karp and Gilliam strains following acute infection in mice
Source: PLoS Negl Trop Dis. 2023 Dec 13;17(12):e0011445. doi: 10.1371/journal.pntd.0011445 (PMC10752558; doi:10.1371/journal.pntd.0011445)
Supplement: S1 Table — (DOCX) [file pntd.0011445.s001.docx]

| **Supplementary table 1** | | | |
| --- | --- | --- | --- |
|  |  |  |  |

**Real-time PCR primers of mouse genes**

**Forward (5’ to 3’) Reverse (5’ to 3’)**

*Cxcl1* CTGGGATTCACCTCAAGAACATC CAGGGTCAAGGCAAGCCTC

*Cxcl2* CCAACCACCAGGCTACAGG GCGTCACACTCAAGCTCTG

*Ccl3* TTCTCTGTACCATGACACTCTGC CGTGGAATCTTCCGGCTGTAG

*Ccl4* TTCCTGCTGTTTCTCTTACACCT CTGTCTGCCTCTTTTGGTCAG

*Ccl5* GCTGCTTTGCCTACCTCTCC TCGAGTGACAAACACGACTGC

*Ifng* AACGCTACACACTGCATCTTGG GCCGTGGCAGTAACAGCC

*Mincle* AGTGCTCTCCTGGACGATAG CCTGATGCCTCACTGTAGCAG

*Ccl2* TTAAAAACCTGGATCGGAACCAA GCATTAGCTTCAGATTTACGGGT

*Fcgr1* AGGTTCCTCAATGCCAAGTGA GCGACCTCCGAATCTGAAGA

*Mx2* GAGGCTCTTCAGAATGAGCAAA CTCTGCGGTCAGTCTCTCT

*Egr2*  GCCAAGGCCGTAGACAAAATC CCACTCCGTTCATCTGGTCA

*Il1b* GCAACTGTTCCTGAACTCAACT ATCTTTTGGGGTCCGTCAACT

*Tnf* CCCTCACACTCAGATCATCTTCT GCTACGACGTGGGCTACAG

*Gapdh* AGGTCGGTGTGAACGGATTTG TGTAGACCATGTAGTTGAGGTCA
