# Supplementary figures and images for: Differential cellular immune responses against Orientia tsutsugamushi Karp and Gilliam strains following acute infection in mice
Source: PLoS Negl Trop Dis. 2023 Dec 13;17(12):e0011445. doi: 10.1371/journal.pntd.0011445 (PMC10752558; doi:10.1371/journal.pntd.0011445)

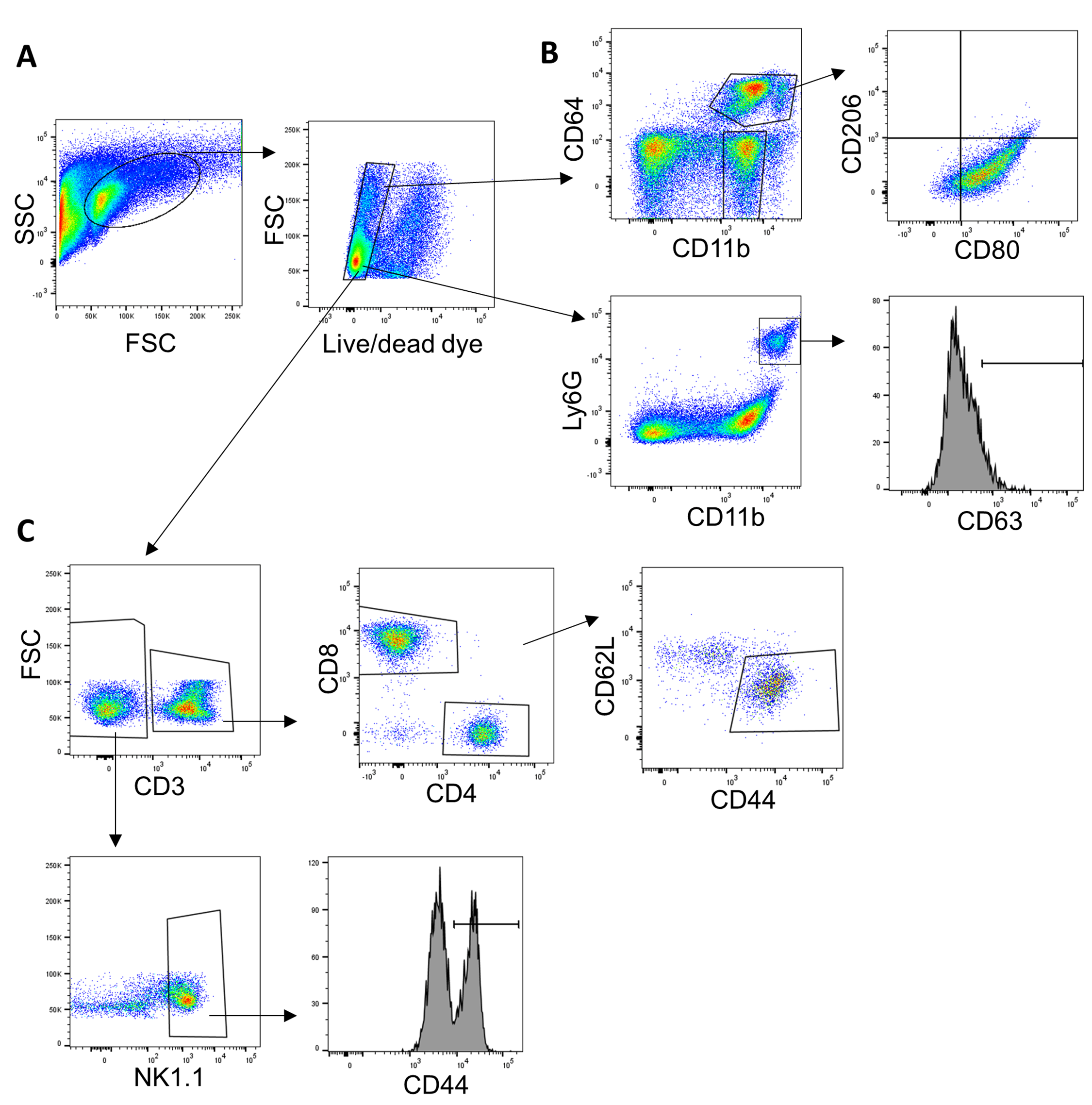

Supplement: S1 Fig — A) Lymphocytes were gated according to FSC and SSC, respectively. Live/dead dye-negative cells were identified as live cells. B) Macrophages were gated on CD11b+CD64+ cells. CD80+CD206- macrophages were characterized as M1 macrophages. Monocytes were characterized as CD11bintCD64-cells. CD11bhiLy6Ghi subpopulation was identified as neutrophils, with CD63 as the neutrophil activation marker. C) Total T cells were gated on CD3 first, followed by CD4 and CD8 gating. CD44+CD62L- T cells were considered activated T cells. CD3-NK1.1+ cells were identified as NK cells, with CD44 as the activation marker. (TIF) [file pntd.0011445.s003.tif]

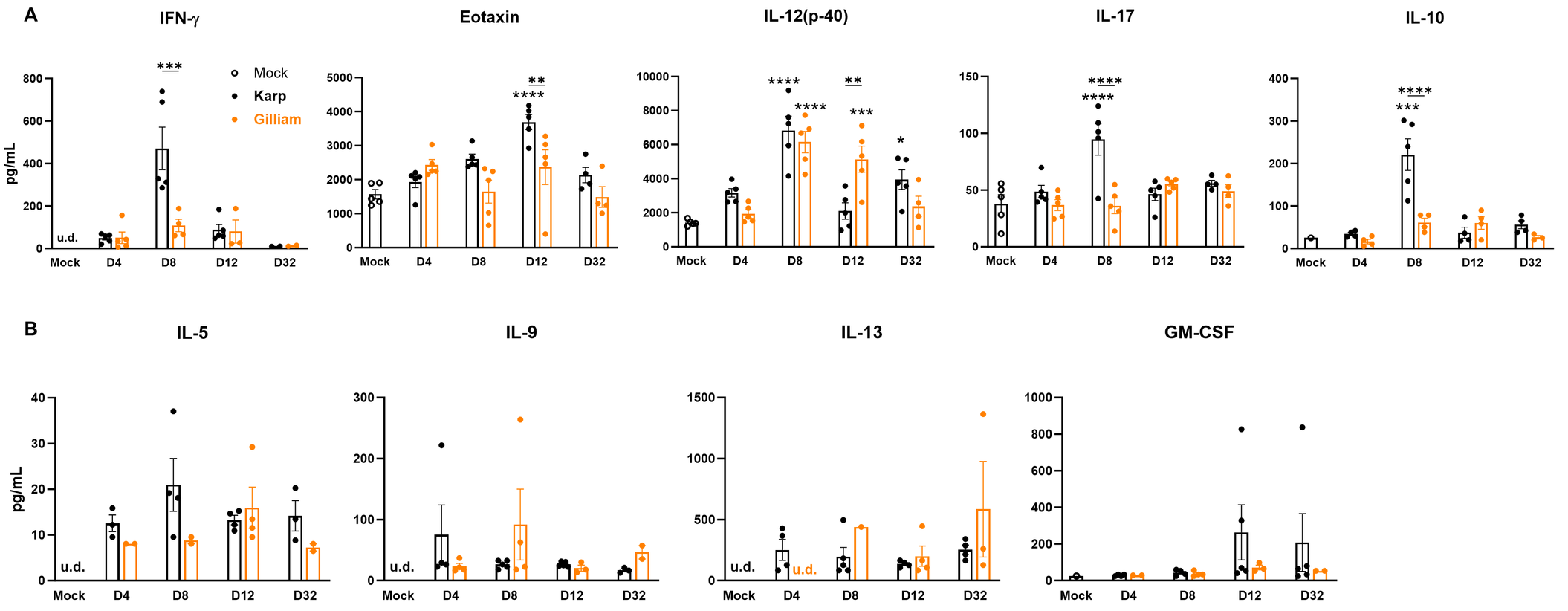

Supplement: S2 Fig — B6 mice were infected, as described in Fig 1. Whole blood was collected for serum preparation at days 4, 8, 12, and 32 and used for cytokine/chemokine measurement via a Bioplex assay (5 mice/group). A) Protein levels show significant difference between Karp- and Gilliam-infected mice in one of two independent studies. B) Protein levels show no significant difference between two strains. Data are presented as mean ± SEM. One-way ANOVA was used for statistical analysis. Šídák’s multiple comparisons test was used for multiple comparison (asterixis above brackets). Dunnett’s multiple comparisons test was used for the comparison of each infected group to mocks (asterixes above columns). *, p < 0.05; **, p < 0.01; ***, p < 0.001; ****, p < 0.0001. (TIF) [file pntd.0011445.s004.tif]
